# The Species of Gut Bacteria Associated with Antitumor Immunity in Cancer Therapy

**DOI:** 10.3390/cells11223684

**Published:** 2022-11-19

**Authors:** Xiaoqiang Qi, Yajun Liu, Samira Hussein, Grace Choi, Eric T. Kimchi, Kevin F. Staveley-O’Carroll, Guangfu Li

**Affiliations:** 1 Department of Surgery, University of Missouri-Columbia, Columbia, MO 65212, USA; 2 School of Medicine, University of Missouri-Columbia, Columbia, MO 65212, USA; 3 Ellis Fischel Cancer Center, University of Missouri-Columbia, Columbia, MO 65212, USA; 4 Department of Molecular Microbiology and Immunology, University of Missouri-Columbia, Columbia, MO 65212, USA

**Keywords:** gut microbiota, antitumor, immunotherapy, probiotics, fecal microbiota transplantation

## Abstract

Both preclinical and clinical studies have demonstrated that the modulation of gut microbiota could be a promising strategy for enhancing antitumor immune responses and reducing resistance to immunotherapy in cancer. Various mechanisms, including activation of pattern recognition receptors, gut commensals-produced metabolites and antigen mimicry, have been revealed. Different gut microbiota modulation strategies have been raised, such as fecal microbiota transplantation, probiotics, and dietary selection. However, the identification of gut bacteria species that are either favorable or unfavorable for cancer therapy remains a major challenge. Herein, we summarized the findings related to gut microbiota species observed in the modulation of antitumor immunity. We also discussed the different mechanisms underlying different gut bacteria’s functions and the potential applications of these bacteria to cancer immunotherapy in the future.

## 1. Introduction

Recently, the association between gut microbiota and antitumor immunity has been widely acknowledged. Both preclinical and clinical studies have demonstrated gut microbiota modulation could be a promising strategy for enhancing the effectiveness of cancer immunotherapies. The gut microbiota affects antitumor immunity in multiple ways. The function of pattern recognition receptor (PRR) signaling was the first to be recognized. PRRs, as lipopolysaccharides (LPS) produced by gut bacteria, can activate Toll-like receptor 4 (TLR4), thus inducing a series of inflammatory reactions to influence the development of liver cancer [1]. Our group’s study observed that TLR9 was also involved in the gut microbiota-mediated antitumor immune response in liver cancer. Secondly, antigens from some specific gut bacteria species can induce an antigen-specific immune response, which cross-reacts with the tumor-associated antigen (TAA) to suppress tumor progression. For instance, *Enterococcus hirae* produce antigens that have similar epitopes in terms of structure with TAA, thus inducing CD4^+^ T-helper type 1 (Th1) cell responses in advanced lung cancer patients treated with chemo-immunotherapy [2]. Furthermore, gut bacteria-derived metabolites play important roles in the modulation of antitumor immunity. For instance, butyrate produced by *Faecalibacterium prausnitzii* was associated with an improved clinical response to the treatment of immune checkpoint blockade (ICB) against cancer [3]. The antitumor immune response involves different immune cell subsets including antigen-presenting cells (APC), CD4^+^ Th1 cells, CD8^+^ T cells, CD4^+^ T regulatory cells (Treg), and myeloid-derived suppressor cells (MDSC) [4,5,6,7]. Specifically, the interferon-γ-producing effector CD8^+^ T cells were induced by the supplementation of a consortium consisting of 11 bacterial strains that resulted in enhanced antitumor immunity. Mechanistically, the increased frequency of T cells were associated with bacterially mediated chronic recruitment, cellular expansion, and bacterial antigen-induced differentiation [8]. To effectively modulate gut microbiota in the treatment of cancer, different approaches have been developed, such as probiotics, dietary supplementation, and fecal microbial transplantation (FMT). FMT aims to transfer the fecal material from an identified donor to a recipient, thus reshaping the gut microbiota profile in the recipient for the activation of antitumor immunity. A phase 1 clinical trial (NCT03353402) assessed the safety, feasibility, and function of FMT in patients with metastatic melanoma. The data demonstrated that FMT effectively exerted favorable changes in immune cell infiltration and gene expression in the tumor microenvironment and improved clinical outcomes in 30% of the patients treated with ICB [9]. 

All these findings convince us that the modulation of the gut microbiota could be a potential strategy for improving responses to cancer therapy. To develop the gut microbiota-based treatment for cancer, the identification of a targeted gut bacteria species is required [10]. This review summarized the current findings related to the specific gut bacteria species involved in the modulation of antitumor immunity in both preclinical and clinical investigations. The different gut bacteria species were classified as “favorable bacteria”, “unfavorable bacteria”, and “bacteria associated with either favorable or unfavorable effects on antitumor immunity in different contexts”. Although a large amount of evidence was provided by many exciting investigations, it is still difficult to define a “good” or “bad” gut bacteria species for cancer treatment because of the lack of sufficient data, the conclusions in different observations are even controversial. The field requires not only more experiments designed with more strict controls and in more specific scenarios, but also more cellular and molecular mechanism studies to support each conclusion. Moreover, in terms of the complexity of gut bacteria commensal with thousands of bacteria species, perhaps targeting a gut microbial consortium instead of a single gut bacteria species could constitute a better strategy in cancer treatment, which may lead us to the future direction of this field. 

## 2. Bacteria Species Associated with Favorable Modulation in Antitumor Immunity

### 2.1. Bifidobacterium spp.

*Bifidobacterium* is a genus of Gram-positive anaerobic bacteria; it may be the most well-known probiotic because of its friendly inhabitation of the human gut. The growth of *Bifidobacterium* strains in the gut can expel other non-helpful microorganisms so as to keep people healthy. Early in 2015, Sivan et al. reported that *Bifidobacterium* has favorable properties for the antitumor immune response. In a preclinical study, they found that using mice purchased from different facilities to establish tumor models resulted in different tumor growth rates with distinct tumor-infiltrating lymphocyte profiles. The mice from Jackson Laboratory had a slower tumor growth rate than the mice from Taconic Farm using the same procedures. This difference can be abolished by cohousing the mice. More interestingly, a FMT from Jackson donor mice to Taconic recipient mice before tumor inoculation could improve effector CD8^+^ T cell infiltration into the tumor and retard tumor progression in the recipient mice. A gut microbiota analysis via 16s ribosomal RNA gene sequencing showed that the *Bifidobacterium* operational taxonomic unit (OTU) is 400 times more abundant in Jackson mice than Taconic mice [11]. 

A recent study reported that *Bifidobacterium* facilitates CD47-based immunotherapy through accumulation in the tumor microenvironment (TME). The systemic administration of mixed species of *Bifidobacterium* led to the accumulation of *Bifidobacterium* spp. in the tumor, which reversed the resistance of anti-CD47 immunotherapy in a colon cancer mouse model. The mechanistic study showed that in the mice deprived of the stimulator of interferon genes (STING), which regulates the expression of type I interferon (IFN), the administration of *Bifidobacterium* could not sufficiently enhance the antitumor activity produced by anti-CD47. This proved that *Bifidobacterium* worked together with STING in the dendritic cells (DCs) to enhance the antigen presentation on DCs, overcoming the CD47 signal and accumulating more T cells [12].

In the consideration of gut microbiota as a large microbial ecosystem, some researchers proposed that the modulation of an individual gut microbiota species is not enough to improve the efficacy of immunotherapy for cancer or reverse the resistance towards immune response; perhaps a modulation targeting a group of gut microbiota species will be more feasible for altering the response to the treatments. However, researchers have found an increase in *Bifidobacterium* could mediate the entire commensal community. One investigation associated *Bifidobacterium* with a change in the other microbial species in the gut, such as an increased level of *Lactobacillus*. It helps ameliorate adverse effects induced by cytotoxic T-lymphocyte-associated protein 4 (CTLA4) blockade. Mechanistically, unlike cells containing Treg, Treg-depleted cells have shown that there was no increase in the gut microbiota species number in the presence of *Bifidobacterium*. This led to the understanding that *Bifidobacterium* alter the microbiota composition in conjunction with Treg cells [13]. 

In a clinical study, researchers found more abundant *Bifidobacterium* in patients with melanoma who responded to a treatment of programmed cell death protein 1 (PD1) blockade compared to patients who failed to respond to PD1 blockade [14]. The *Bifidobacterium* species *B. longum* was reported to be enriched in patients with non-small cell lung carcinoma (NSCLC) who responded to anti-PD1 treatment accompanied by higher levels of memory CD8^+^ T cells and natural killer (NK) T cells in their blood [15]. Even given the lack of direct evidence and mechanistic studies, these findings suggest *Bifidobacterium* are beneficial for the antitumor immune response. On the other hand, it has been indicated that *Bifidobacterium* spp. help ameliorate anti-CTLA4 immunotherapy-induced colitis. Administration of *Bifidobacterium* reduced the weight loss caused by CTLA4 blockade accompanied with decreased inflammatory cytokine levels without compromising anti-CTLA4’s therapeutic effect in a mouse model [16].

Despite all the data above, the challenge remains to be the further identification of different *Bifidobacterium* strains, since most studies were performed at the level of the gut microbiota genus, as well as the illustration of the underlying molecular mechanisms. In a study using two strains of *Bifidobacterium breve* (JCM92 and Bb03) collected from two different sources, the significant genetical differences between these two *B. breve* strains resulted in differences in the outcome of an antitumor treatment. Similar to the scenario above, although both strains enhanced the antitumor immune response, e.g., increased T cell activation, only the JCM92 strain boosted the activity of the chemotherapy drug oxaliplatin, CTLA4 blockade, and programmed death-ligand 1 (PD-L1) blockade. However, further analysis is needed to determine the mechanisms behind this observation. Studying the genes of *B. breve*, it was seen that the JCM92 strain but not Bb03 had genes that stimulated RNA, amino acid, and amino-sugar metabolic processes, which are known to contribute to antitumor activities through acid-degrading enzymes [17]. In another study, investigators observed that *Bifidobacterium* strains produce inosine. Inosine, a metabolite of the human body with multiple functions, has a critical role in immune activation. Inosine greatly improves the expression of tumor antigens, allowing cytotoxic immune cells to quickly identify and eradicate tumor cells. The study demonstrated that the presence of inosine increased the level of IFN-γ and TNF-α, which further increased tumor antigen presentation and aided T cell activity. Additionally, studies in a mouse model showed that inosine acted on adenosine 2A receptor on T lymphocytes. Together, inosine and adenosine 2A receptor along with cAMP-PKA induced Th1 cell differentiation in the TME. This mechanism was aided by the increased IL-12Rβ2 and IFN-γ transcription as a response to the phosphorylation of cAMP response element-binding protein (pCREB). In addition, inosine was also involved in macrophage-mediated antibody production [18]. 

### 2.2. Enterococcus hirae

*Enterococcus* is a genus of Gram-positive and facultative anaerobic bacteria. *Enterococcus hirae (E. hirae)* was found to be a favorable gut bacterium for ICB cancer treatment. Clinically, it was shown to be more abundant in cancer patients with a better response to ICB immunotherapy. In a murine model, the recolonization of *E. hirae* could reverse the treatment resistance induced by FMT in non-responding patients [19]. *E. hirae* has been reported to be involved in cyclophosphamide (CTX)’s anticancer effects; also, the translocation of *E. hirae* from the gut into the lymphoid organs induced the generation of T helper 17 (Th17) cells and immune response against tumor [20,21]. In a further study by Daillere et al., an oral administration of *E. hirae* restored the efficacy of CTX in antibiotic (ATB)-treated-sarcoma-bearing mice by increasing the intra-tumoral CD8/Treg ratio [2]. In addition, *E. hirae* induced CD4^+^ Th1 cell responses accompanied with a longer progression-free survival (PFS) in advanced lung and ovarian cancer patients treated with chemo-immunotherapy [2]. This is most likely because *E. hirae* produced antigens possessing similar epitopes in structure with TAA in cancer patients [22]. Recently, the underlying molecular mechanisms were further investigated, wherein a multifaceted mode was defined by which *E. hirae* affected antitumor immunity and enabled anticancer effects of CTX, including inducing IFNγ-producing and CD137-expressing effector memory T cell responses, increasing the local delivery of polyamines, and enriching *Bifidobacteria* in the host [23].

Clinically, both circulating and liver/tumor-infiltrating *E. hirae*-reactive CD8^+^ T cell responses were observed only in HBV-related hepatocellular carcinoma (HCC) patients but not in healthy individuals, and the frequency of these cells was positively corelated with the PFS time of the HCC patients. Mechanistically, the *E. hirae*-associated immune response may suppress the induction of Foxp3^+^ regulatory T cells and PD-1^+^ CD8^+^ T cells [24]. 

*E. hirae* was also recently reported to play a role in the antitumor effect of T-cell immunoglobulin and mucin domain-3 (Tim-3) blockade—Tim-3 is an immune check point protein [25]. The oral gavage of *E. hirae* restored the antitumor efficacy of Tim-3 blockade, which had previously been attenuated by an antibiotic treatment in the preclinical cancer model. 

### 2.3. Ruminococcaceae (Oscillospiraceae) Family 

*Ruminococcaceae* is a family of strictly anaerobic bacteria that are normally present in the colonic mucosal biofilm of healthy individuals [26]. In a study by Panebianco et al. employing a pancreatic ductal adenocarcinoma (PDAC) mouse model, a significant reduction of tumor volume mediated by gemcitabine therapy was related to a reduced proportion of *Ruminococcaceae* from 39 to 17% [27]. However, more studies demonstrated that *Ruminococcaceae* may play a favorable role in the response to immunotherapy. One study analyzed the stool samples from 38 patients with solid tumors treated with anti-PD1, wherein a significant increase in *Ruminococcaceae* was observed in the stool samples from patients who responded to the treatment [28]. Similarly, in another investigation, when applying a FMT from stool samples enriched with *Lachnospiraceae*, *Ruminococcaceae*, and *Veillonellaceae* to nivolumab (anti-PD1 antibodies)-refractory patients, a tumor suppression response was detected in certain partial patients [9]. Furthermore, in a study examining the microbiota of patients with metastatic melanoma who were treated with anti-PD1 immunotherapy, researchers found that the patients responsive to the treatment had a greater relative abundance of *Ruminococcaceae,* in addition to other species, when compared to that in the non-responsive patients [29]. Mechanistically, the analysis of the systemic immune response demonstrated that the patients with a higher abundance of *Ruminococcaceae* in their guts had a higher frequency of effector CD4^+^ and CD8^+^ T cells in circulation and a preserved cytokine production ability [30].

### 2.4. Faecalibacterium spp.

*Faecalibacterium* is a Gram-positive, anaerobic genus of bacteria belonging to the *Ruminococaccaceae* family. It is featured as one of the main species of bacteria in the gut producing short-chain fatty acids (SCFA) through dietary fiber fermentation. *Faecalibacterium* spp. were implicated in a variety of studies focusing on the relationship between the gut microbiota and cancer [30]. In the study mentioned above [29], the patients with metastatic melanoma responding to anti-PD1 antibodies (anti-PD1 Abs) had a higher relative abundance of *Faecalibacterium* compared to that in non-responsive patients. The responders also had a longer PFS accompanied by greater effector CD8^+^ T cells tumor infiltration. This study concluded that the abundance of *Faecalibacterium* in the fecal microbiota is a strong microbial predictor for a clinical response to anti-PD1 therapy, along with the alpha diversity and the abundance of *Bacteroidales* [29]. In another clinical study, 26 melanoma patients who received anti-CTLA4 Abs treatment were investigated; consequently, *Faecalibacterium* spp. presented in a higher proportion in patients who had a better response to the treatment and the longer PFS and overall survival (OS). Analysis of the fecal microbiota of metastatic melanoma patients receiving ipilimumab (anti-CTLA4 Abs) revealed that the enriched *Faecalibacterium* were associated with a longer survival, but also an increased occurrence of ipilimumab-induced colitis [31]. 

*Faecalibacterium prausnitzii* (*F. prausnitzii*) is a key butyrate producer with multifaceted roles in inflammatory responses, as it has been associated with an improved clinical response to the treatment of ICB but also functions to mitigate intestinal inflammation in the context of inflammatory bowel disease [3]. *F. prausnitzii* is able to produce SCFAs; a recent study revealed that SCFAs actually promoted cellular metabolism, enhanced the memory potential of activated CD8^+^ T cells, and were required for the optimal recall responses upon antigen re-encounter [32]. Gopalakrishnan et al. reported an enrichment of *Faecalibacterium* spp. in fecal samples from melanoma patients responding to PD-1 blockade [29]. The study by Peters et al. revealed that the presence of *Faecalibacterium* spp. in pre-treatment stool samples was correlated with a longer PFS of melanoma patients receiving immunotherapy [33]. Another clinical investigation by Botticelli et al. demonstrated in NSCLC patients treated with nivolumab that *Faecalibacterium* was more abundant in the feces of responders than that in the non-responders [34]. 

### 2.5. Oscillibacter spp.

*Oscillibacter* is a genus of Gram-negative and anaerobic bacteria belonging to the *Ruminococcaceae* family. A preclinical study analyzing the effects of gut microbiota modulation on HCC growth revealed that tumor growth was significantly suppressed when the model mice were fed with a diet of “Prohep”, a probiotic mixture. In addition, the tumor suppression was accompanied by altered angiogenesis and antitumor immune responses. The analysis of gut microbiota profiles identified the significant enrichment of several gut microbiota species including *Oscillibacter* spp. in treated mice. This study showed that the increase in *Oscillibacter* together with *Prevotella* reduced tumor-infiltrating Th17 cells [35,36]. In a gut microbiota analysis of patients with gastric cancer and gastrointestinal stromal tumors (GIST), a lower abundance of *Oscillibacter* together with *Lactobacillaceae* were observed in cancer patients compared to healthy controls [37]. Controversially, another clinical investigation revealed that patients with colorectal cancer (CRC) presented an increased mucosal microbiota abundance of *Oscillibacter* together with *Bacteroides*, *Roseburia*, and *Ruminococcus*. However, no mechanistic study was mentioned; thus, the finding needs further verification [38,39]. 

### 2.6. Burkholderia spp. 

*Burkholderia* is a genus of Gram-negative and obligately aerobic gut bacteria. It has been reported that the recolonization of *Burkholderia* spp. in antibiotically treated mice or germ-free (GF) mice could restore anti-CTLA4 Abs’ therapeutic effect against metastatic melanoma. In this investigation, the researchers observed that a diversified gut microbiota and *Burkholderia* specifically were required for anti-CTLA4-mediated antitumor effects, in which using antibiotically treated mice or GF mice could abolish the antitumor response to CTLA4 blockade [40]. In addition, *Burkholderia pseudomallei* was used for a modified carrier of an antitumor vaccine because of its size, shape, and inherent expression of pathogen-associated molecular patterns and invasion-assistant adhesion proteins. Engineered *Burkholderia pseudomallei* loaded with tumor lysates and CpG enhanced DC maturation and TAA cross-presentation, thereby inducing cellular and humoral antitumor responses and suppressing tumor growth in tumor models [41].

### 2.7. Prevotella spp.

*Prevotella* is a genus of Gram-negative anaerobic gut bacteria. *Prevotella copri* was studied for its correlation with rheumatoid arthritis. Recently, it was found to be related to the therapeutic effect of immunotherapy against NSCLC. The clinical data demonstrate that together with two other bacteria, *Prevotella copri* was enriched in patients who responded to anti-PD1 treatment accompanied with higher levels of memory CD8^+^ T cells and NKT cells in the blood [15]. 

Recently, the contribution of the gut microbiota to castration-resistant prostate cancer (CRPC) was studied. The defined gut microbiota facilitated castration resistance in mice, and these bacteria in mice and patients with CRPC were associated with the function of converting androgen precursors into active androgens. An FMT from hormone-sensitive prostate cancer patients and a *Prevotella stercorea* administration suppressed tumor progression [42].

## 3. Bacteria Species Associated with Unfavorable Modulation in Antitumor Immunity

### 3.1. Fusobacterium nucleatum

*Fusobacterium nucleatum* (*F. nucleatum*) is a Gram-negative anaerobic bacillus that has reservoirs in the human mouth, gastrointestinal tract, and other areas. *F. nucleatum* is a well-known pathogenic bacterium [43,44] that has often been isolated from different types of infectious samples collected from patients. The once understudied bacterial strain has proven to be not just opportunistically infectious but also a contributor to tumorigenesis [45]. It has been implicated in various types of cancer, including colorectal cancer, esophageal cancer, gastric cancer, head and neck squamous cell carcinoma, pancreatic cancer, and hepatocellular carcinoma [10].

In multiple studies of CRC, *Fusobacterium* strains have been detected as a potential biomarker for CRC. In addition, the data demonstrated that the presence of *F. nucleatum* in CRC cells was not stage-dependent; it could be potentially detected in cancer cells from stage 0 to IV [46]. In a clinical study of CRC patients, *F. nucleatum* promoted chemoresistance in an oxaliplatin treatment through the activation of the innate immune system [47]. In another study by Flanagan, enriched *F. nucleatum* was observed in stool samples from CRC patients compared to healthy controls [48]. In addition, Mima et al. observed that the enrichment of *F. nucleatum* was associated with worse clinical outcomes in CRC patients [49]. *F. nucleatum* was reported to promote tumor development by inducing inflammation through activation of tumor-associated neutrophils / M2 macrophages and inhibition of cytotoxicity of T and NK cells that repressed the host immune responses [50]. In fact, *Fusobacterium* aids tumorigenesis through multiple pathways. The surface protein FadA on *F. nucleatum* binds to E-cadherin presented on CRC and non-CRC cells, stimulating β-catenin signaling and thus causing inflammation and oncogenicity in the cells. Normal E-cadherin functions to suppress tumors by attaching cells together and reducing motility. However, after binding with FadA, E-cadherin loses its function, and the tumor cells grow and metastasize. This also allows *F. nucleatum* to enter the epithelial and CRC cells, and with the aid of its ability to feed on glucose and amino acids, *F. nucleatum* survives in the cells without challenges regarding nutrient sources. In addition, *F. nucleatum* biofilms have been seen in CRC cells due to the adhesive nature of *F. nucleatum,* which can successfully form biofilms while carrying out the respiration process in hypo-toxic situations. Furthermore, an autophagy mechanism was activated by *F. nucleatum* that could promote cancer cell survival and potentially induced chemoresistance [47,51,52]. Moreover, *F. nucleatum* has also been associated with D-galactose-β (1–3)-N-acetyl-D-galactosamine (Gal-GalNAc) overexpression in cancerous cells. A study has shown that the Fap2 protein on *F. nucleatum* can bind to Gal-GalNAc, which contributes to the increased number of *F. nucleatum* in CRC, thereby supporting further tumorigenesis [53]. The T cell immunoreceptor with Ig and ITIM domains (TIGIT) is a receptor presented on T and NK cells. The inhibition of the TIGIT can suppress NK cell cytotoxicity. The Fap2 protein also functions to bind to the TIGIT to inhibit NK cell-induced cytotoxicity, aiding cancer cells to survive from immune attack [54]. All the above suggests that the abundance of *F. nucleatum* might be an early marker of CRC. In addition, antibiotics targeting *F. nucleatum* could be a safeguard for people with potential risk of CRC. 

### 3.2. Escherichia coli

*Escherichia coli* (*E. coli*) is a Gram-negative and facultative anaerobic gut bacteria species belonging to class *Gammaproteobacteria*. Most *E. coli* are harmless to the host, but some serotypes may cause poisoning. It has been illustrated that *Gammaproteobacteria* can hinder the effects of chemotherapy on the tumors. For instance, Gemcitabine (2′,2′-difluorodeoxycytidine), a chemotherapeutic drug, is used to treat patients with pancreatic, lung, breast, or bladder cancers, but *Gammaproteobacteria* strains that produce the bacterial enzyme cytidine deaminase (CDD_L_) can significantly metabolize the gemcitabine to its inactive form, 2′,2′-difluorodeoxyuridine, to trigger drug resistance. To establish this, a study compared tumor cells treated with an *E. coli* strain that expressed CDD_L_ or with a CDD_L_-deficient *E. coli* strain. The results confirmed the role of the CDD_L_-expressing *E. coli* in inducing resistance to gemcitabine [55]. 

Additionally, the *E coli* strain has a gene called “pks” coding genotoxin colibactin, a polyketide-peptide that causes DNA damage. A study has illustrated that even at low doses, live pks^+^ *E. coli* induced short-lived DNA damage that contributed to the anaphase bridges and chromosome abnormalities caused by insufficient DNA repair mechanisms. Colibactin or colibactin-producing bacteria alter the TME so as to encourage the formation of senescent cells, which help with tumor promotion and cancer progression via the production of growth factors. To clarify, investigators introduced pks^+^ and pks^−^ *E. coli* into intestinal cells, and the cells infected with pks^+^ had an increased number of growth factors stimulating tumor growth. In short, pks^+^ *E. coli* cells had an increased level of senescence-associated β-galactosidase (SA-β-gal) activity that induced the senescence of intestinal epithelial cells, which produced growth factors that contributed to tumor growth. *E coli* downregulated the expression of SENP1, a protein that regulates the sumoylation pathway. The sumoylation of cells has been known to contribute to cell senescence. Upon studying the effect of pks^+/−^ *E. coli* in colorectal tumors, it was established that the tumor cells and TME had an increased number of hepatocyte growth factor (HGF) mRNAs, activated HGF receptor, some senescence markers such as SA-β-gal and p21cip, and a reduction in the number of SENP1-expressing cells. This result supports the finding that pks gene-containing *E. coli* assists in tumorigenesis asserted in previous studies [56,57]. 

Furthermore, in a clinical investigation, researchers observed that *E. coli* were more abundant in patients with melanoma who did not respond to anti-PD1 treatment than in patients who responded well to the treatment. In addition, the patients with more *E. coli* had a shorter PFS accompanied with a higher degree of tumor infiltration of Treg cells [29]. 

### 3.3. Ruminococcus spp.

*Ruminococcus* is a genus of Gram-positive anaerobic bacteria recently found in the human gut that belongs to *Ruminococcaceae* family. The study by Matson et al. involving 42 metastatic melanoma patients receiving a treatment of PD-1 blockade demonstrated that *Ruminococcus obeum* were over-presented within the microbiota of the poor responders [14]. In addition, in NSCLC patients, Botticelli et al. reported that *Ruminococcus bromii* were less presented in the responders treated by nivolumab [34]. Recently, a study with 27 metastatic melanoma patients receiving immunotherapy revealed that the reduced survival probably was related to the over-presented *Ruminococcus gnavus* [33]. In another clinical study in China, the gut microbiota profiles from patients with NSCLC receiving anti-PD1 Abs treatment were analyzed; the results demonstrated that *Ruminococcus* spp. were mainly found in non-responding patients. However, the defined correlation and mechanisms behind this observation require further investigation [15]. 

### 3.4. Gammaproteobacteria Class

*Gammaproteobacteria*, mentioned above, is a large class of bacteria that has been implicated in the regulation of the therapeutic efficacy of some anticancer drugs. A study employing a colon cancer mouse model observed that the chemotherapy drug gemcitabine was converted into its inactive form by the bacterial enzyme cytidine deaminase, an enzyme seen primarily in *Gammaproteobacteria* [55]. Therefore, gemcitabine resistance was induced by intratumor *Gammaproteobacteria* and ameliorated by antibiotic treatment. As gemcitabine is often used for the treatment of PDAC, the researchers found an increased level of *Gammaproteobacteria* in the pancreatic tumors compared to the normal pancreatic tissues and culturing the bacteria from fresh PDAC tumors with human colon carcinoma cell lines rendered the cell lines fully resistant to gemcitabine. These results led the researchers to hypothesize that the presence of *Gammaproteobacteria* was a key factor in the metabolism of gemcitabine and a possible target for tumors’ sensitization to gemcitabine treatment. However, it is still unclear whether *Gammaproteobacteria* can impact antitumor immunity in PDAC patients treated with gemcitabine. 

## 4. The Bacteria Species Associated with Both Favorable and Unfavorable Modulation in Antitumor Immunity

### 4.1. Akkermansia muciniphila

*Akkermansia muciniphila* (*A. muciniphila*) is a Gram-negative, strictly anaerobic bacterium and a minor constituent in the intestinal microbiota. It was proposed to be associated with many diseases including inflammation, obesity, diabetes, and even cancer. A recent study demonstrated that the administration of *A. muciniphila* exacerbated tumor growth in a preclinical colorectal cancer model accompanied with an increased expression of tumor cell proliferation-associated gene profile [58]. Consistently, *A. muciniphila* was identified as one of four gut bacteria, which, as a biomarker panel, were significantly over-represented in patients with colorectal cancer compared to healthy people in a clinical investigation [59]. 

However, *A. muciniphila* has also recently been recognized as a favorable gut bacteria species for ICB treatments against cancer. The metagenomics analysis of clinically collected fecal samples from NSCLC and renal cell carcinoma (RCC) patients revealed that *A. muciniphila* was significantly abundant in the patients with the best clinical results. More interestingly, an FMT from ICB-non-responding patients could induce resistance to ICB treatment in GF mice, but the recolonization of *A. muciniphila* could reactivate the therapeutic effect of ICI in these mice via an interleukin-12-dependent manner [19]. Furthermore, Panebianco *et al.* noted that gemcitabine, an FDA-approved chemotherapy drug for PDAC, induced a reduction in tumor volume (approximately 35%) in a PDAC mouse model depended on the shift of *A. muciniphila* from 5 to 33% in gut bacterial composition [27]. Xu et al. analyzed the correlation between the treatment of different antibiotics and the efficacy of the anti-PD1 antibody in CT26 tumor-bearing colorectal carcinoma model mice. *A. muciniphila* was enriched in the vancomycin-treated group, which was associated with an increased benefit of anti-PD-1 therapy. This development indicated that *A. muciniphila* maintains the normal efficacy of anti-PD1 antibody by affecting the metabolism of glycerophospholipids [60]. In another study by Botticelli et al., the fecal samples from NSCLC patients who responded to the treatment of nivolumab were more abundant in *A. muciniphila* [61]. Recently, a prospective study by Salgia et al. collected fecal samples from 31 metastatic RCC patients before they received immunotherapy for a gut microbiota analysis. The results revealed that the enrichment of *A. muciniphila* was related to the patients’ clinical benefit from an ICB treatment [62]. *A. muciniphila* has also been implicated in the modulation of abiraterone acetate (AA) therapy’s efficacy, which is used for prostate cancer. *A. muciniphila* utilized AA as an energy source and inhibited *Corynebacterium* growth; this shift in the gut microbiota improved the therapeutic efficacy of AA, but determining the further underlying mechanisms warrants more research [63,64].

### 4.2. Bacteroides spp.

*Bacteroides* is a genus of Gram-negative, obligate anaerobic bacteria. Clinically, *Bacteroides species* (*B.* spp.) are considered significant pathogens and are found in most anaerobic infections when they escape from the gut, but they are beneficial for the host when they remain in the gut [65]. For instance, *B. thetaiotaomicron* (*B.th*) are typically associated with healthy people due to their important role in the acquisition and utilization of different carbohydrates [66,67]. 

An immunological study has reported that a zwitterionic polysaccharide (ZPS) produced by *B. fragilis* could activate CD4^+^ T cells via presentation on antigen-presenting cells (APC). The data demonstrated that the ZPS from *B. fragilis* was important in the development of CD4^+^ T cells, and GF mice present a lower proportion of CD4^+^ cells, which could be corrected by recolonization with *B. fragilis* [68,69]. The results from several studies have suggested a close relationship of *Bacteroides* and the immune system. In a recent investigation involving cancer immunotherapy, *Bacteroides* was determined to be required for the response to the CTLA4 blockade against melanoma/NSCLC in both human patients and murine models [40]. Initially, investigators found that the treatment of CTLA4 Abs often resulted in an adverse effect on gut immune homeostasis [70]. Later on, it was found that CTLA4 Abs disturbed the intestinal bacterial flora, causing the dysbiosis of gut immunity. Further studies demonstrated that gut bacterial flora significantly impacted the therapeutic efficacy of CTLA4 Abs. Experiments using a mouse tumor model showed that the antitumor effect of CTLA4 Abs can be abolished by an antibiotic cocktail or the use of GF mice, which suggested that gut bacterial flora were required for the efficacy of CTLA4 Abs against tumors. Furthermore, *B.th* and *Bacteroides fragilis* (*B.f*), but not the other species in the *Bacteroides* genus, were identified to be capable of recovering the response to a CTLA4 Abs treatment via recolonization in GF mice or antibiotic treated mice. The underlying mechanisms require further study to reveal the key features in *B.th* and *B.f* that are different from others. Nonetheless, the above findings were supported by the analysis of the gut microbiota composition in melanoma patients treated with ipilimumab [40]. 

Moreover, both the prevalence and the relative abundance of *B. salyersiae* were higher in responders versus non-responders in the stool of RCC patients treated with nivolumab, and the efficacy of nivolumab was successfully restored in nonresponding RCC-bearing mice after a compensation with an oral administration of *B. salyersiae* [71]. Frankel et al. demonstrated that more *B.th* presented in the responders to ipilimumab plus nivolumab therapy in patients with metastatic melanoma [72]. 

Despite the cases above suggesting *Bacteroides* spp. as favorable bacteria for antitumor immunity, some contrary findings have been reported. In a clinical study with 26 melanoma patients who received anti-CTLA4 Abs treatment, *Bacteroides* spp. were more abundant in non-responders [31]. From a metagenomic analysis of 112 melanoma patients by Gopalakrishnan et al., the abundance of *B.th* was correlated with poor responses to PD-1 blockade, and patients with higher levels of *Bacteroidales* have a reduced survival rate [29]. Furthermore, a recent study by Peters et al. analyzed the pre-treatment stool samples from 27 melanoma patients undergoing immunotherapy; the gut microbiota data revealed that *B. dorei*, *B. massiliensis,* and *B. ovatus* were related to a shorter survival of patients, in which the pathways for the biosynthesis of 6-hydroxymethyl dihydropterin diphosphate, coenzyme A, flavin, guanosine nucleotides, pantothenate, pyridoxal 5-phosphate, and the degradation of L-rhamnose were involved [33]. One of the *Bacteroides* spp., enterotoxigenic *Bacteroides fragilis* (ETBF), was specifically studied as a disease-causing *Bacteroides*. ETBF has been implicated in the development of CRC. A couple of studies noted that the presence of *Bacteroides fragilis* toxin (BFT) not only activated proinflammatory responses and induced gut microbiota dysbiosis but also contributed to CRC development [73]. Mechanistically, it has been established that ETBF stimulated immunosuppressive responses in TME; the BFT in association with IL-17 converted the harvested myeloid cells to MDSC followed by the other subsequent mechanisms, which helped the tumor cells escape from host immune surveillance [74].

In the consideration of the complex factors involved in the correlation of gut microbiota and cancer development/cancer treatment including cancer types, cancer stages, the context of investigation, and the background of the subjects, we believe both studies showing *Bacteroides* spp. as a favorable gut bacteria and studies showing it as an unfavorable gut bacteria bring us a step closer to a complete understanding of the role of *Bacteroides* spp. in the antitumor immune response. However, more specific criteria for the subjects in the data analysis and further mechanistic studies in each investigation are still required. 

### 4.3. Clostridiales (Eubacteriales) Order and Clostridium spp.

*Clostridiales* is an order of Gram-positive, obligately anaerobic bacteria. A pilot study with rectal cancer patients investigating the association between the gut microbiota composition and therapeutic responses to neoadjuvant chemoradiotherapy (nCRT) observed a significant enrichment of *Clostridiales* in non-responders [75]. Vetizou et al. found that in melanoma patients receiving immunotherapy with ipilimumab, the gut microbiota was modified towards a relative increase in *Clostridiales* [40], but how this change feedbacked to the effect of ipilimumab is still not clear. 

Although implicated in pathogenic infections of the gut, the genus *Clostridium* under the order *Clostridiales* was found to be elevated in a variety of cancers; however, the possibility of using *Clostridium* in cancer therapy has been discussed in several studies. An investigation demonstrated that *Clostridium* aided in CD8^+^ T cell expansion in the gut and distal organs, promoting an effective response to immunotherapy in patients with NSCLC [76]. In addition, patients with a higher abundance of *Clostridium* and *Lactobacillus* in their stool tended to have longer PFS and OS benefits from immunotherapy than the patients with a lower abundance of *Clostridium* [77]. 

### 4.4. Klebsiella spp.

*Klebsiella* is a genus of Gram-negative, facultative anaerobic bacteria. *Klebsiella pneumoniae* (*K. pneumoniae*) is the most significant species in this genus and it acts as a pathogen in nosocomial infections. Recently, *K. pneumoniae* were found to be responsible for nonalcoholic fatty liver disease (NAFLD), which is one of the important causes of the initiation of hepatocellular carcinoma [78]. The investigators identified that *K. pneumoniae* was strongly associated with endogenous alcohol production in a patient with severe nonalcoholic steatohepatitis (NASH); they also found that *K. pneumoniae* overgrowth in the gut microbiota could represent a critical pathogenesis in NAFLD patients, which was verified in a murine model [78]. A recent clinical investigation involving 110 patients with metastatic CRC demonstrated that *Klebsiella quasipneumoniae* exhibited the top upregulation in abundance in patients who had a progressive disease under the targeted chemotherapy versus the patients who had responses to the treatment [79]. 

However, a favorable role of *K. pneumoniae* in clinical cancer immunotherapy has been studied: Matson et al. studied the association of the microbiota composition and the responses to PD-1 blockade in 42 metastatic melanoma patients and found that *K. pneumoniae* were more abundant in the fecal samples from responders [40].

### 4.5. Alistipes spp.

*Alistipes* is a relatively new genus that was isolated from human gut microbiota. It has been correlated with cancer development and treatment [80]. A clinical study collecting the data from NSCLC patients who received anti-PD1 Abs treatment demonstrated that *Alistipes putredinis* together with another two bacteria were enriched in the responding patients, which was accompanied with a higher level of memory CD8^+^ T cells and NKT cells in the periphery blood [15]. A recent metagenomic analysis of the gut microbiota observed a reduced relative abundance of *Alistipes onderdonkii* in an orthotopic, patient-derived xenograft model; the data in vitro demonstrated that *Alistipes onderdonkii* was able to inhibit pancreatic tumor cells’ proliferation and suppress the growth of pancreatic primary cancer cells [81]. 

However, in another study, an increased abundance of *Alistipes* spp. was observed in the nipple aspirate fluid of breast cancer patients, but the causative relationship requires further study [39].

## 5. Prospects

Although studies have unveiled the above gut microbiota bacteria species’ involvement in the modulation of antitumor immunity with different roles, more evidence and further mechanistic studies are required to define them as favorable (Table 1) or unfavorable (Table 2) gut bacteria in the context of cancer treatment. First, many clinical reports analyzed the gut microbiota profiles of patients with different clinical outcomes from the same procedures, based on which the association of specific gut bacteria species and antitumor immune responses was statistically concluded; indeed, this provided significant, valuable data, but also left a large space for the further verification and explanation of the various mechanisms. Second, with regard to the complexity and heterogeneity of cancers, every definition must follow a strict context or scenario, which has been reflected in the sections of this review. Some gut bacteria were found to both enhance and inhibit the antitumor immune response (Table 3), which may depend on the cancer type, disease stage, and patient’s background. For instance, *Bacteroides* spp. were observed to play either beneficial or unbeneficial roles in different clinical investigations involving melanoma patients, which leads us to consider the distinct backgrounds of these patients. Lastly, reviewing the same gut bacteria studied in different investigations, various mechanisms were proposed for the observations, which suggests to us that gut bacteria-mediated antitumor immune modulation most likely acts through multiple pathways. For example, *Bifidobacterium* spp. is well-known probiotics that benefit antitumor immune responses in cancer treatments. However, different mechanisms were elucidated including effector CD8^+^ T cell tumor infiltration [11], the production of inosine [18], and the maintenance of host microbiota diversity [13]. 

Recently, the use of advanced computing techniques in the identification of favorable/unfavorable gut bacteria in cancer treatments has dramatically facilitated the accumulation of acknowledge in this field. A metagenomic analysis of gut microbiota data and treatment outcomes from patients with eight different cancer types revealed that responder patients had a significantly higher microbial diversity and different microbiota compositions compared to non-responders. Specific species, *Bacteroides ovatus* and *Bacteroides xylanisolvens*, were screened out by a machine-learning model and validated in a preclinical murine model for their positive correlation with treatment outcomes [82]. Another machine-learning meta-analysis of 16S rRNA gene-sequencing data from a mixed tumor patient cohort and three published gut microbiome datasets from melanoma patients identified the gut bacterial taxa associated with a response to immunotherapy regardless of the tumor type [83]. These findings support the development of gut microbiota-based cancer treatments. 

Despite the insufficient number of mechanistic studies, the rapidly accumulating evidence for the gut bacterially mediated improvement of antitumor immune responses encourages us to progress towards a translational study to apply antitumor-favorable bacteria in cancer therapy. Basically, the current gut microbiota modulation strategies include FMT, probiotics, and diet-based selection, all of which aim to reshape the patients’ gut microbiota commensally with a higher abundance of favorable bacteria and a lower abundance of unfavorable bacteria. In addition, *Mycobacterium bovis* Bacillus Calmette-Guérin (BCG) has been used as the gold-standard treatment specifically for non-muscle-invasive bladder cancer (NMIBC) in clinics since the 1970’s. BCG, as a bacterially based intravesical immunotherapy, could induce a robust antitumor immune response involved in effector CD8^+^ T cells and NKT cells’ infiltration without toxicity [84,85]. In consideration of the important role of bile acids (BAs) in the development and treatment of liver cancer by modulating hepatic lipid and glucose metabolism [86,87,88,89], the gut bacteria participating in BA metabolism have been studied for improving antitumor immune responses in HCC treatment. The data demonstrated that the removal of Gram-positive bacteria by antibiotics, which contains the bacteria mediating the primary-to-secondary bile acid conversion, was sufficient for inducing hepatic NKT cell accumulation and suppressing liver tumor growth [89,90,91]. 

As mentioned, *E. hirae* is involved in the favorable anticancer effects of CTX chemotherapy [23], while *Gammaproteobacteria* strains, such as *E. coli*, can hinder the tumor-killing effects of gemcitabine [55], which could be leveraged for improving the chemotherapeutic efficacy against tumors. Meanwhile, the effects of chemotherapeutic drugs on the gut microbiota should be considered in clinical practice. CTX has been demonstrated to reduce the abundance of *Firmicutes* phylum bacterial species, *lactobacillus* species, and *enterococcus* species in the small intestine of a preclinical tumor model [21]. Gemcitabine was reported to reduce Firmicutes and Bacteroidetes and, conversely, increase *Proteobacteria* and *Verrucomicrobia* (*Akkermansia muciniphila*) levels in a mouse model [27]. The interaction of gut bacteria and chemo-drugs could be an important factor in the development of gut microbiota-based antitumor immunomodulatory strategies in the chemotherapeutics field. 

In conclusion, despite the insufficient description of the mechanisms and the safety concerns associated with gut microbiota-based therapeutic approaches in cancer treatment, the FMT, prebiotics, diet-based selection, or combined strategies show important potential with respect to curing cancer. It is necessary to define the role of each gut bacteria species in cancer treatments; furthermore, the identification of a gut microbiota profile for each patient may become a routine examination procedure in precision medicine and personalized medicine in the future. 

## Figures and Tables

**Table 1:** The bacteria associated with favorable modulation in antitumor immunity.

Bacteria	Research Objects	Interventions	Identified Mechanism
*Bifidobacterium* spp.	Colon cancer murine model	CD47-based immunotherapy	STING pathway and type I IFN production [12]
	Patients with melanoma	Anti-PD1 Abs	Unknown [14]
	Patients with NSCLC	Anti-PD1 Abs	CD8^+^ T cells and NKT cells [15]
*Enterococcus hirae*	Sarcoma murine model	Cyclophosphamide	CD4 Th1 cells [2]
*Ruminococcaceae* (family)	PDAC mouse model	Gemcitabine	Unknown [27]
	Patients with metastatic melanoma	Anti-PD1 Abs	Unknown [29]
	Patients with solid tumors	Nivolumab	Effector CD4^+^ and CD8^+^ T cells [30]
*Faecalibacterium* spp.	Patients with metastatic melanoma	Anti-PD1 Abs	Effector CD8^+^ T cell tumor infiltration [29]
	Patients with melanoma	Anti-CTLA4 Abs	Short chain fatty acid (SCFA) & butyrate [30]
	Patients with NSCLC	Nivolumab	Unknown [34]
*Oscillibacter* spp.	HCC murine model	N/A	Tumor infiltrating Th17 cells [35,36]
	Patients with gastric cancer	N/A	Unknown [37]
*Prevotella* spp.	Patients with NSCLC	Anti-PD1 Abs	CD8^+^ T cells and NKT cells [15]
*Alistipes* spp.	Patients with NSCLC	Anti-PD1 Abs	CD8^+^ T cells and NKT cells [15]
*Burkholderia* spp.	Metastatic melanoma murine model	Anti-CTLA4 Abs	Unknown [40]

**Table 2:** The bacteria associated with unfavorable modulation in antitumor immunity.

Bacteria	Research Objects	Interventions	Identified Mechanism
*Ruminococcus* spp.	Patients with metastatic melanoma	Anti-PD1 Abs	Unknown [14]
	Patients with NSCLC	Anti-PD1 Abs	Unknown [34,40]
*Gammaproteobacteria*(Glass)	Colon cancer murine model	Gemcitabine	Bacterial enzyme cytidine deaminase [55]
	PDAC murine model	Gemcitabine	Gemcitabine metabolism [55]
*Fusobacterium nucleatum*	Patients with CRC	Oxaliplatin	tumor-associated Neutrophils & M2 macrophages [50]
*Escherichia coli*	Patients with melanoma	Anti-PD1 Abs	Treg cells tumor infiltration [29]

**Table 3:** The bacteria observed with both favorable and unfavorable modulation in antitumor immunity.

Bacteria	Favorable Role			Unfavorable Role		
	Research Objects	Interventions	Identified Mechanism	Research Objects	Interventions	Identified Mechanism
*Akkermansia muciniphila*	Patients with NSCLC and RCC	Anti-PD1 Abs	Interleukin 12-dependent [19]	CRC murine model	N/A	Proliferation-associated gene upregulation [58]
	PDAC murine model	Gemcitabine	Unknown [27]	Patients with CRC	N/A	Unknown [59]
	CRC murine model	Anti-PD1 Abs	Glycerophospholipid Metabolism [60]			
	Patients with NSCLC	Nivolumab	Unknown [61]			
*Bacteroides* spp.	Patients with NSCLC and murine model	Anti-CTLA4 Abs	ZPS production [68,69]	Patients with melanoma	Anti-CTLA4 Abs	Unknown [31]
	Patients with RCC	Anti-PD1 Abs	Unknown [71]	Patients with melanoma	Anti-PD1 Abs	Unknown [29]
	Patients with metastatic melanoma	Anti-CTLA4 Abs	Unknown [72]	Patients with CRC	N/A	MDSC [74]
*Clostridium* spp.	Patients with NSCLC	ICB	Unknown [77]	Patients with rectal cancer	nCRT	Unknown [75]
*Klebsiella pneumoniae*	Patients with metastatic melanoma	Anti-PD1 Abs	Unknown [40]	Patients with HCC	N/A	Endogenous alcohol production [78]
*Alistipes* spp.	Patients with NSCLC	Anti-PD1 Abs	CD8^+^ T cells & NKT cells [15]	Patients with breast cancer	N/A	Unknown [39]

## Data Availability

Not applicable.
